# IL-1Β Enriched Monocytes Mount Massive IL-6 Responses to Common Inflammatory Triggers among Chronically HIV-1 Infected Adults on Stable Anti-Retroviral Therapy at Risk for Cardiovascular Disease

**DOI:** 10.1371/journal.pone.0075500

**Published:** 2013-09-25

**Authors:** Emilie Jalbert, Timothy Q. Crawford, Michelle L. D’Antoni, Sheila M. Keating, Philip J. Norris, Beau K. Nakamoto, Todd Seto, Nisha I. Parikh, Cecilia M. Shikuma, Lishomwa C. Ndhlovu, Jason D. Barbour

**Affiliations:** 1 Hawaii Center for HIV/AIDS, Honolulu, Hawaii, United States of America; 2 Department of Tropical Medicine, John A. Burns School of Medicine, University of Hawaii, Honolulu, Hawaii, United States of America; 3 Blood Systems Research Institute, San Francisco, California, United States of America; 4 Department of Laboratory Medicine, University of California San Francisco, San Francisco, California, United States of America; 5 Department of Medicine, University of California San Francisco, San Francisco, California, United States of America; 6 Department of Medicine, John A. Burns School of Medicine, University of Hawaii, Honolulu, Hawaii, United States of America; 7 Straub Clinics and Hospital, Honolulu, Hawaii, United States of America; 8 Queen’s Medical Center, Honolulu, Hawaii, United States of America; Institute of Infectious Diseases and Molecular Medicine, South Africa

## Abstract

Chronic infection by HIV increases the risk of cardiovascular disease (CVD) despite effective antiretroviral therapy (ART). The mechanisms linking HIV to CVD have yet to be fully elucidated. High plasma levels of the pro-inflammatory cytokine IL-6, which may be triggered by IL-1β, is a biomarker of CVD risk in HIV-negative adults, and of all-cause mortality in HIV disease. Monocytes play a pivotal role in atherosclerosis, and may be major mediators of HIV-associated inflammation. We therefore hypothesized that monocytes from HIV-infected adults would display high inflammatory responses. Employing a 10-color flow cytometry intracellular cytokine staining assay, we directly assessed cytokine and chemokine responses of monocytes from the cryopreserved peripheral blood of 33 chronically HIV-1 infected subjects. Participants were 45 years or older, on virologically suppressive ART and at risk for CVD. This group was compared to 14 HIV-negative subjects matched for age and gender, with similar CVD risk. We simultaneously detected intracellular expression of IL-1β, IL-6, IL-8 and TNF in blood monocytes in the basal state and after stimulation by triggers commonly found in the blood of treated, chronically HIV-infected subjects: lipopolysaccharide (LPS) and oxidized low-density lipoprotein (oxLDL). In the absence of stimulation, monocytes from treated HIV-infected subjects displayed a high frequency of cells producing IL-1β (median 19.5%), compared to low levels in HIV-uninfected persons (0.9% p<0.0001). IL-8, which is induced by IL-1β, was also highly expressed in the HIV-infected group in the absence of stimulation, 43.7% compared to 1.9% in HIV-uninfected subjects, p<0.0001. Strikingly, high basal expression of IL-1β by monocytes predicted high IL-6 levels in the plasma, and high monocyte IL-6 responses in HIV-infected subjects. Hyper-inflammatory IL-1β enriched monocytes may be a major source of IL-6 production and systemic inflammation in HIV-infected adults, and may contribute to the risk for all-cause mortality and cardiovascular disease in treated HIV infection.

## Introduction

High blood plasma IL-6 is a biomarker of cardiovascular disease (CVD) risk in adults [1], and is associated with increased overall mortality in adults with HIV-1 infection (HIV+) [2]. HIV+ adults are at elevated risk for developing CVD [3,4], however it remains unclear how HIV infection contributes to this risk and what mechanisms may be involved. Determination of routes by which IL-6 comes to be expressed to high levels in the blood during HIV infection, namely the source of its production and triggers, may shed light on HIV-driven processes that increase risk for all-cause mortality and CVD, and in turn reveal clues in the search for new markers of CVD risk in HIV. Indeed, an effective biomarker of CVD risk that is specific to the HIV+ population has not yet emerged [5]. IL-6 is one candidate biomarker, among several.

Studies based on the SMART (Strategies for Management of Anti-Retroviral Therapy) trial indicate that HIV-1 infected patients who initiate and remain on anti-retroviral therapy (ART) show sustained reductions in levels of inflammatory agents such as IL-6, C-Reactive Protein (CRP) and D-Dimer, as well as improvements in high-density lipoprotein (HDL) and low-density lipoprotein (LDL) levels, and their respective transport proteins ApoA1 and ApoB [5]. However the degree of improvement of HDL-particle (HDL-p) and ApoA1 levels was lower in those with high baseline CRP and IL-6, indicating a relationship between high chronic immune inflammation and an impaired ability to resolve dyslipidemia in chronic HIV infection. The authors suggest that the beneficial effects of ART on dyslipidemias may be driven by reductions in immune inflammation. Understanding the mechanisms behind elevated chronic immune inflammation in HIV, and in particular its relation to IL-6 levels in the blood, may inform strategies to target immune inflammation and persistent dyslipidemias that contribute to CVD risk and other sources of morbidity [6] and mortality in chronic HIV infection.

Recently, studies of HIV chronic inflammation have increasingly focused on blood monocytes, a population of cells with inflammatory properties known to be involved in promotion of atherosclerosis [7,8]. Monocytes are circulating, highly secretory cells that respond to a wide range of stimuli, including microbial products and oxidized lipoproteins. These cells represent an important source of cytokines and chemokines and may be major contributors to systemic immune inflammation in HIV disease. Monocytes produce IL-1β a pleiotropic cytokine having multiple and diverse inflammatory properties [9]. Indeed, IL-1β is considered the gatekeeper of inflammation [10], and triggers many secondary responses, including IL-6 production in monocytes [11].

Upon stimulation, monocytes synthesize the immature IL-1β precursor, which is cleaved by caspase-1 into its mature form. In circulating human monocytes, caspase-1 is present in an already active form, as opposed to macrophages, which require inflammasome activation to produce active caspase-1 [12]. Many endogenous stimulants have been shown to participate in the production of IL-1β, such as IL-1β itself, activated complement, uric acid crystals, high concentration of glucose, cholesterol, free fatty acids and oxidized free fatty acids, such as oxidized LDL (oxLDL). Circulating blood monocytes, due to their innate sensing role and ability to rapidly secrete IL-1β in response to stimuli, may play a major role in IL-6 production in HIV-1 infected adults.

We developed a protocol to detect simultaneous expression of four cytokines, IL-1β, IL-8 (CXCL8), IL-6 and TNF, in viably preserved monocytes [13]. This approach has several advantages over ELISA based detection of monocyte supernatants, western blotting, mRNA species detection or prior monocyte flow based assays (4 colors or less). Notably, our assay provides detailed monocyte phenotyping, allowing localization of response by monocyte subsets. Inclusion of four cytokines in a single panel allows characterization of polyfunctionality.

We chose two commonly encountered inflammatory triggers in the blood as stimuli to evaluate the inflammatory responsiveness of blood monocytes: 1) lipopolysaccharide (LPS), a bacterial product that is elevated in the blood of many HIV+ persons and a known contributor to immune inflammation during HIV disease and 2) oxidized low density lipoprotein (oxLDL), a species of LDL associated with inflammation and found at elevated levels in the blood of adults at risk for CVD. We hypothesized that monocyte populations from chronically HIV-infected subjects on virologically suppressive ART with CVD risk factors, would exhibit high basal levels of IL-1β, and hyper-inflammatory, polyfunctional monocyte responses to LPS and oxLDL, including high production of IL-6 and other cytokines.

## Materials and Methods

### Cohort description

Our observational cohort study consisted of 33 chronically infected HIV+ subjects on ART, drawn from a parent cohort study of HIV and cardiovascular disease, above a median of 50 years of age, and 14 HIV-uninfected subjects recruited into the same parent cohort, intended to be similar with respect age, gender and cardiovascular risk factors (Table 1). Details on the parent cohort have been previously described [14]. Whole blood was drawn into EDTA tubes and cells were processed for peripheral blood mononuclear cells (PBMC) isolation within one hour of collection. Plasma was isolated by centrifugation, removed and stored at -80°C whereas cells were isolated using a standardized ficoll-based protocol and cryopreserved in liquid nitrogen storage until use. The University of Hawaii Manoa Committee on Human Studies approved this study, and all subjects gave written informed consent to participate in this study including permission to have blood specimens banked and utilized for future research related to HIV and cardiovascular health.

**Table 1:** Demographic and Clinical Characteristics.

	**HIV-1 Infected (N = 33)**	**HIV-1 Negative (N = 14)**
	**Median (IQR) or Percent**	**Median (IQR) or Percent**
**Age in Years**	53 (49-56)	51 (46-60)
**Systolic Blood Pressure (mm Hg)**	120 (116-130)	119 (113-133)
**Total Cholesterol (mg/dL)**	175 (146-189)	173 (151-192)
**LDL (mg/dL)**	101 (81-122)	107 (86-114)
**HDL (mg/dL)**	36 (30-45)*	55 (46-64)
**Body Mass Index**	25.7 (23-27)	24 (22.5-27)
**hsCRP**	1.2 (0.5-1.7)	0.7 (0.4-1.7)
**HIV RNA copies/mL**	48 (48-48)	NA
**CD4+ T cell count (cells/μL)**	574 (450-713)	ND
**CD8+ T cell activation (%CD38/HLA-DR)**	11.7 (8.5-16.7)*	6.9 (4.5-11.3)
**On combination anti-retroviral therapy**	100%	NA
**History of hypertension**	30%	29%
**On antihypertensive therapy**	27%*	7%
**History of High Cholesterol**	48%	43%
**Low HDL**	60%*	14%
**On anti-hyperlipidemia therapy**	27%*	14%
**Current smoker**	33%	29%
**Male**	87%	100%
**Caucasian**	67%	64%

* p < 0.05

IQR = interquartile range, NA = Not Applicable, ND = Not Determined, LDL = low-density lipoprotein, HDL = high-density lipoprotein, hsCRP = high sensitivity C reactive protein

### Monocyte Intracellular Cytokine Staining (Mono-ICS) Assay

Cryopreserved PBMC were thawed in serum-free media (AIM *V* *Medium* , Life Technologies) containing 10 µg/ml of DNAse (Sigma) rested overnight at 37°C and 5% CO_2_ in a polypropylene 96-well plate. The next day, the cells were stimulated with either oxLDL (10µg/ml), LPS (100ng/ml) or media alone (unstimulated) for 6 hours in the presence of brefeldin-A (5µg/ml) and monensin (5µg/ml). Cells were then surface-stained with CD3 (V500), CD14 (Qdot605), CD16 (Alexa700), CD56 (PE-Cy7), CD19 (PE-Cy7), CD20 (PE-Cy7), HLA-DR (APC-H7) antibodies, and with Live/Dead fixable yellow dead cell stain (yellow amine reactive dye: YARD). Cells were subsequently fixed, permeabilized (BD FACS Lyse, Perm Buffer II) and stained with conjugated antibodies against IL-1β (PE), IL-8 (FITC), IL-6 (APC) and TNFα (PerCP-Cy5.5). All antibodies are from BD Biosciences, except for CD14 Q605 and Live/Dead fixable yellow dead cell stain (Life Technologies) and TNFα (eBiosciences).

### Flow Analysis

Data was acquired on a custom 4-laser BD LSRFortessa, and all compensation and gating analyses were performed in FlowJo (TreeStar).

### Plasma Inflammatory Factor Detection

Plasma samples were assayed for IL-6 using antibody coated beads in a high-sensitivity Milliplex assay (Human CVD panel, EMD Millipore, Billerica, MA). Standard curves and samples were tested in duplicate. Samples were acquired on a Labscan 200 analyzer (Luminex, Austin, Tx) using Bio-Plex manager software (Bio-Rad, Hercules, CA).

### Statistical Analysis and Data Presentation

We employed non-parametric statistical tests (Mann-Whitney U for comparison tests, and Spearman Rank test for correlations). Measures of central tendency were expressed as median with interquartile range (median, IQR). We applied the Bonferroni correction to instances of multiple-comparisons testing, determined as p = 0.05/X, where X represents the number of comparisons. For example, in the case of the Boolean gate analysis we divided the p-value threshold of 0.05 by 16 (the number of comparisons made per condition), rendering a new, adjusted p-value threshold of 0.003. All significance results in this report were adjusted for multiple comparisons as needed, and only those results meeting the adjusted significance threshold are reported as a numeric value, with those tests not passing Bonferroni adjustment reported simply as ‘NS’, or are not listed. Statistical analyses were performed in GraphPad/Prism for Mac OS X. Presentation of distributions was performed using GraphPad/Prism and SPICE version 5.1, downloaded from http://exon.niaid.nih.gov [15].

## Results

Clinical and demographic characteristics of the subjects included in this analysis are listed in Table 1. All patients receiving ART were administered a regimen of at least three anti-retroviral agents including two nucleoside reverse transcriptase inhibitors (nRTI) and a non-nucleoside reverse transcriptase inhibitor or a ritonavir boosted protease inhibitor (PI). HIV-infected subjects were of similar median age, and had similar rates of hypertension, total cholesterol, LDL, high sensitivity CRP (hsCRP), and current smoking rates compared to HIV-uninfected subjects. HIV-infected subjects had lower HDL, higher CD8+ T cell activation and greater rates of treatment for hypertension and cholesterol than HIV-uninfected subjects.

### Gating strategy to identify total monocytes, monocyte subsets and cytokine production

**Figure 1:**
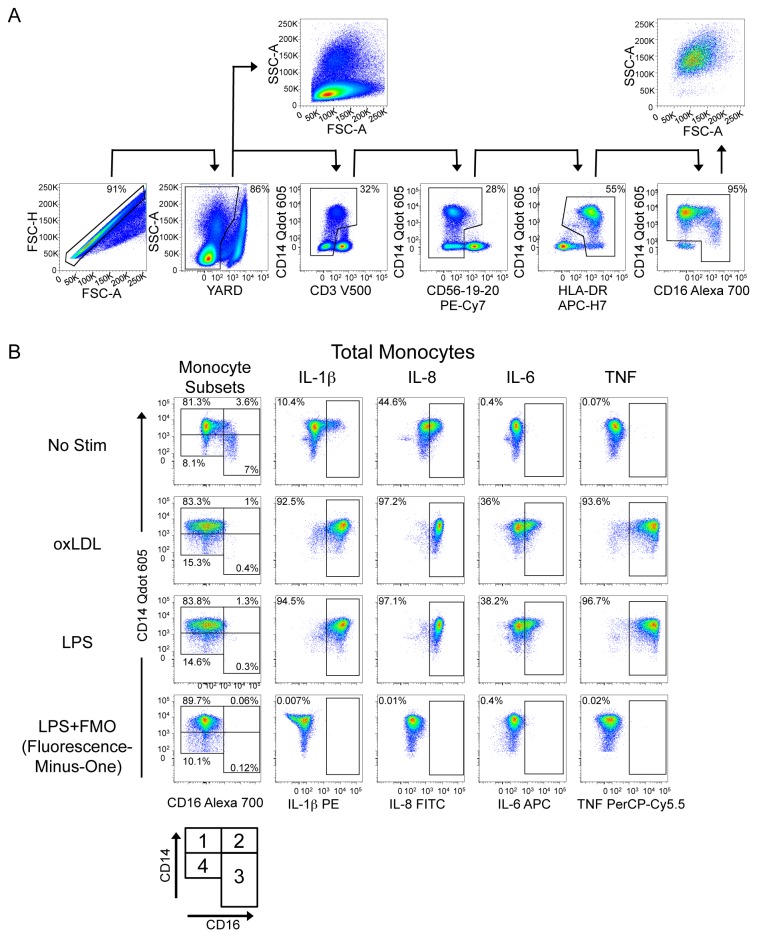
Gating strategy for identification of total monocytes, monocyte subsets and detection of cytokine expression. Gating Strategy. Panel A. Identification of total monocytes from peripheral blood mononuclear cells by exclusion of doublets, dead cells, CD3, CD56, CD19, CD20 and low HLA-DR expressing cells. Double-negative CD14-CD16- cells were also excluded. Forward scatter (FSC) vs. side scatter (SSC) plots are shown comparing total PBMC (doublet and dead cells excluded) and total monocytes. Panel B. Monocyte subsets were identified based on the expression of CD14 and CD16 (left column). The diagram at the bottom of the left column is a visual guide for the terminology of monocyte subsets employed in this report. Intracellular cytokines (IL-1β, IL-8/CXCL8, IL-6 and TNF) produced in total monocytes were detected in response to no stimulus, oxidized low density lipoprotein (oxLDL) or lipopolysaccharide (LPS). Fluorescence minus one control condition, in which the antibody conjugate in question is omitted to guide creation of the gate that defines positive expression of that target, is shown on the bottom row. The subject presented is HIV-infected and displays high but representative responses to stimuli.

Monocytes were identified within total PBMC by excluding doublets, dead cells, CD3, CD56, CD19, CD20 and HLA-DR low expressing cells (Figure 1A). We categorized monocytes into 4 populations (Figure 1B), termed Mono 1-4 for simplicity. Mono 1, 2 and 3 have been previously characterized as the classical monocytes (Mono1, CD14++CD16-), intermediate monocytes (Mono2, CD14++CD16+) and non-classical monocytes (Mono3, CD14+/CD16+). It is believed that non-classical monocytes represent a more mature subset, originating from the classical subset and transitioning through the intermediate subset [16,17,18]. In our assay, we also observed a population of CD14+ CD16- monocytes, characterized by reduced but still detectable levels of CD14, that we termed Mono4. We excluded double negative CD14-CD16- cells since these are typically classified as dendritic cells [19,20]. Figure 1B (right 4 columns) displays the gates used to determine positivity for IL-1β, IL-8, IL-6, and TNF in total monocytes.

### Monocytes from HIV-infected subjects exhibit greater inflammatory cytokine responses

**Figure 2:**
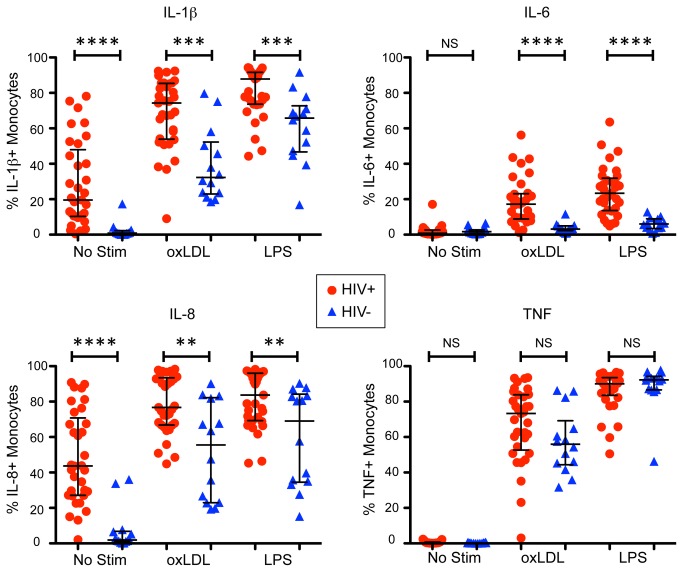
Monocyte production of pro-inflammatory cytokines in the basal state and upon stimulation. HIV-infected subjects are shown in red circles and HIV-uninfected subjects are shown in blue triangles. In the no stimulation condition (basal state), HIV-infected subjects showed higher levels of IL-1β (upper left panel) and IL-8 (lower left panel). Upon stimulation with either oxLDL or LPS, HIV-infected subjects exhibited higher levels of IL-1β, IL-8 and IL-6 (upper right panel) compared to the HIV-uninfected subjects. While HIV-1 infected subjects did tend to have higher TNF (lower right panel) responses upon stimulation, these differences were not significant. **** p < 0.0001, *** p < 0.001, ** p < 0.01.

Under the basal, unstimulated condition, monocytes from HIV-uninfected subjects produced little to no detectable levels of any cytokine examined (Figure 2, Table 2). In contrast, monocytes from HIV-infected subjects exhibited high basal production of both IL-1β and IL-8, but not IL-6 or TNF. Upon stimulation with oxLDL or LPS, monocytes from both the infected and uninfected groups produced IL-1β, IL-8 and TNF, however the HIV-infected subjects showed significantly higher frequencies of IL-1β or IL-8 expressing monocytes compared to the HIV-uninfected group. There was no statistically significant difference between the HIV-infected and HIV-uninfected groups with respect to TNF responses to oxLDL or LPS.

**Table 2:** Frequency (percent) of monocytes producing specific cytokines.

		IL-1β	IL-8	IL-6	TNF
No Stim	HIV+	19.5 (10.3-48.0)	43.7 (27.1-70.8)	0.8 (0.4-2.6)	0.3 (0.1-0.7)
	HIV-	0.9 (0.2-2.3)	1.9 (1.0-6.8)	1.7 (0.6-2.7)	0.2 (0.1-0.3)
oxLDL	HIV+	74.3 (53.9-85.4)	76.7 (66.8-93.5)	17.2 (8.9-23.2)	73.3 (52.7-83.9)
	HIV-	32.3 (23.0-52.3)	55.5 (23.0-82.2)	3.1 (2.3-5.0)	56 (44.4-69.2)
LPS	HIV+	87.9 (73.6-91.7)	83.7 (69.3-96.0)	23.4 (13.6-31.9)	90 (83.6-93.5)
	HIV-	65.8 (46.7-72.7)	69.1 (34.6-84.2)	6.1 (3.5-8.9)	92.3 (86.7-94.3)

Data is shown as median (25^th^ percentile - 75^th^ percentile). This table summarizes data displayed in Figure 2.

Strikingly, IL-6 was substantially induced in the HIV-infected subjects upon stimulation with either oxLDL or LPS, whereas the HIV-uninfected subjects showed minimal induction. This was not due to higher basal IL-6 levels in the HIV-infected subjects since both the HIV-infected and HIV-uninfected groups had very low frequencies of IL-6 expressing cells in the unstimulated condition.

### Polyfunctional, inflammatory monocytes are increased in HIV-1 infected adults

**Figure 3:**
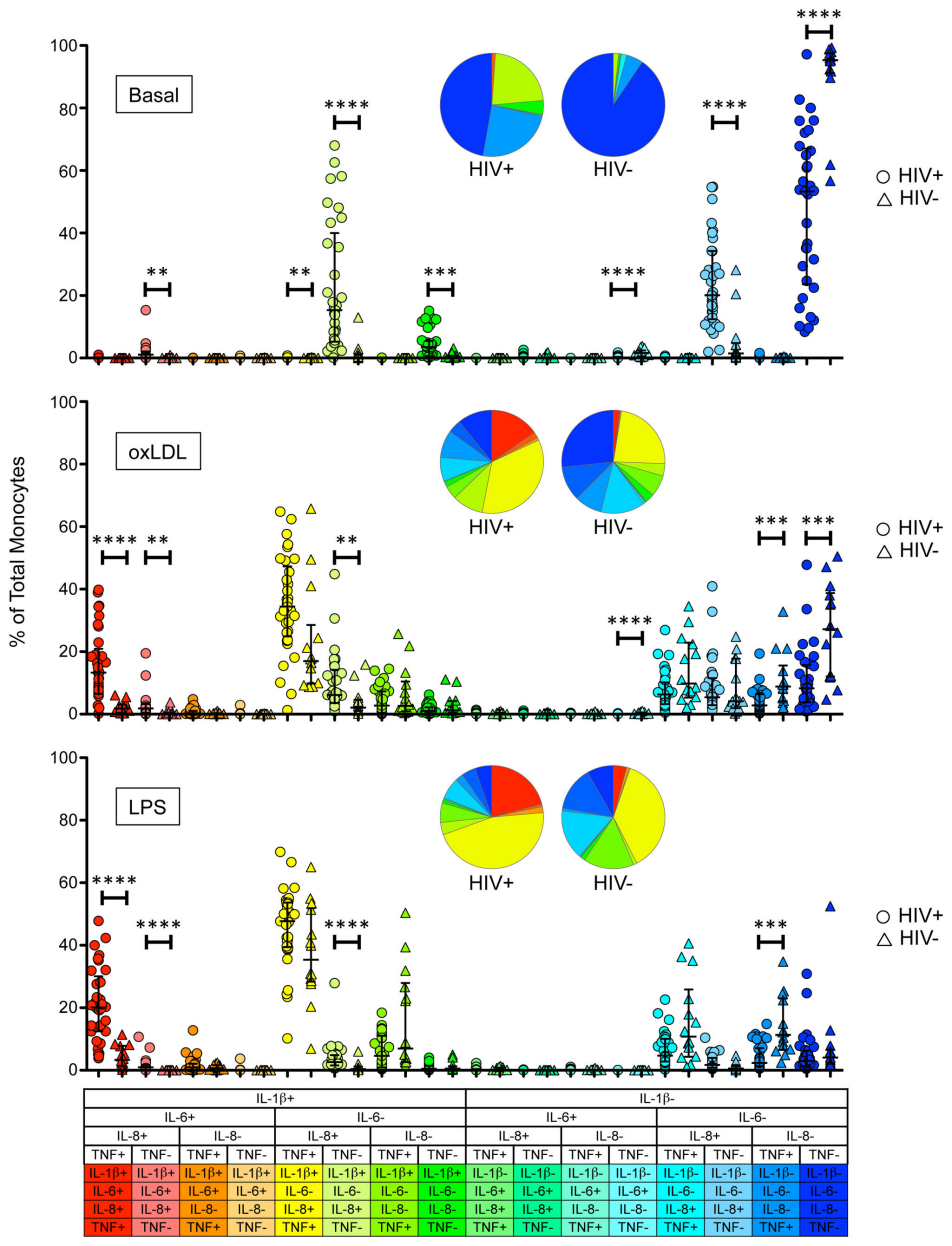
Induction of broad, high frequency, polyfunctional monocyte responses to stimuli. HIV-infected subjects are shown in circles, HIV-uninfected are shown in triangles. Top Panel: Unstimulated (basal) state, Middle Panel: After oxLDL stimulation, Bottom Panel: After LPS stimulation. To determine the frequency of cells producing one or more cytokines, we performed a sum of the median responses. Mann Whitney U was used for comparison tests, and all comparisons were adjusted for multiple comparisons (p = 0.05/16 comparisons per stimulation condition = p = 0.003), and only p-values meeting or exceeding the p-value threshold are shown. **** p < 0.0001, *** p < 0.001, ** p < 0.003.

In order to determine whether there was production of multiple cytokines within the same cell or if different cells were each producing different cytokines, we performed a combinatorial cytokine analysis using a Boolean approach (Figure 3). This technique allowed us to examine whether individual cells were producing 0, 1, 2, 3 or all 4 cytokines. In the unstimulated condition (Figure 3 top panel), a median of only 4.7% (IQR 2.5-8.8) of monocytes from HIV-uninfected samples produced any combination of cytokines, compared to 46.7% (33.0-76.4) in the HIV-infected group (sum of all responses). Cells producing IL-8 alone (sky blue) represented 20.1% (12.5-34.3) of the total monocytes from HIV-infected subjects, whereas cells producing both IL-8 and IL-1β (yellow-green) represented 15.3% (5.3-40.0) of total monocytes. A smaller fraction (3.5% (0.7-5.5)) produced only IL-1β.

Upon stimulation with oxLDL (Figure 3 middle panel), 72.8% (61.3-88.0) of the monocytes from the HIV-uninfected group responded by producing one or more cytokines, compared to 91.7% (84.3-96.2) for the HIV-infected group. Responsive monocytes from HIV-uninfected subjects were composed mainly of triple-positive IL-1β+IL-8+TNF+ (17% (9.8-28.6)) (yellow), double-positive IL-8+TNF+ (9.8% (5.3-22.9)) (turquoise) or single-positive TNF+ (8.8% (4.1-15.6)) (aqua blue) cells. Monocytes from HIV-infected subjects responded to oxLDL mainly with triple-positive IL-1β+IL-8+TNF+ cells (34.4% (24.9-47.3)) (yellow). However, the most striking difference between groups was the frequency of quadruple-positive IL-1β+IL-8+TNF+IL-6+ cells (red), which represented a median of 13.3% (6.5-20.9) of monocytes in the HIV-infected group, compared to only 1.8% (0.3-3.1) in the HIV-uninfected group. This result indicated that IL-6 was only produced in cells that were also producing other cytokines. Indeed, the cells producing all 4 cytokines provided the single largest source of IL-6.

Upon stimulation with LPS (Figure 3 bottom panel), 95.9% (93.4-97.9) of the monocytes from the HIV-uninfected group produced one or more cytokine, compared to 97.0% (93.7-98.7) for the HIV-infected group. Responding monocytes from HIV-uninfected subjects were composed of triple-positive IL-1β+IL-8+TNF+ (35.4% (28.4-51.9)) (yellow), double-positive IL-8+TNF+ (10.7% (4.3-25.9)) (turquoise) or single-positive TNF+ cells (11.3% (6.6-23.0)) (aqua blue) cells. Monocytes from the HIV-infected group were primarily triple-positive IL-1β+IL-8+TNF+ cells (47.7% (39.5-53.7)) (yellow). Quadruple-positive IL-1β+IL-8+TNF+IL-6+ represented 20.1% (12.5-30.1) (red) in the HIV-infected group, compared to only 3.3% (1.5-7.8) in the HIV-uninfected group. Here too, the cells producing all 4 cytokines provided the single largest source of IL-6. Together these data suggest a potential hierarchy of cytokine production in polyfunctional monocytes, where IL-6 may be preferentially expressed by monocytes that are also producing other cytokines.

### Polyfunctionality and monocyte subset

We then examined the CD14 and CD16 expression profiles of the cytokine producing monocytes to determine which monocyte subset they belonged to (data not shown). In the basal condition, monocytes from HIV-infected subjects that were single-positive for IL-8 were mostly classical (Mono1, CD14++CD16-) monocytes (89.0% (82.7-93.4)). Similarly, of the double-positive IL-8+IL-1β+, 96.2% (93.4-97.2) were Mono1. Single-positive IL-1β+ were also Mono1 (91.0% (81.5-95.3)).

Upon stimulation with either oxLDL or LPS, we observed a strong shift in monocyte subsets compared to the basal state in both the HIV-infected and uninfected groups. Decreases in the frequency of CD16+ subsets, namely the intermediate monocytes (Mono2, CD14+ +/CD16+) and the non-classical monocytes (Mono3, CD14+/CD16+) were seen. Additionally, we observed an increase of frequency in the classical (Mono1 CD14+ +/CD16-) subset and the Mono4 subset (CD14+/CD16-). Since there was no difference in the viability and the overall frequency of total monocytes between the different stimulations, it is possible that cells from the Mono2 subsets lose CD16 expression upon stimulation and are counted as Mono1. A similar scenario is possible for cells in the Mono3 subset that are counted as Mono4 upon stimulation and subsequent loss of CD16 expression.

Under conditions of oxLDL or LPS stimulation, triple-positive IL-1β+IL-8+TNF+ monocytes from HIV-infected subjects were mostly Mono1 (93.3% (89.1-96.1) for oxLDL and 90.6% (86.8-94.7) for LPS), while Mono4 represented a smaller fraction (6.1% (3.4-10.4) for oxLDL and 8.4% (4.4-12.3) for LPS). Double-positive IL-8+TNF+ monocytes were mostly Mono1 (79.3% (56.8-84.3) for oxLDL and 64.2% (56.1-79.8) for LPS) and Mono4 (19.9% (14.4-42.3) for oxLDL and 34.9% (19.5-43.5) for LPS). HIV-uninfected subjects showed similar frequencies. Monocytes from HIV-infected subjects producing all four cytokines were Mono1 (92.9% (86.8-96.5) for oxLDL and 89.3% (84.6-94.6) for LPS) and Mono4 (5.4% (2.5-10.6) for oxLDL and 9.0% (3.7-13.2) for LPS).

### HIV-1 infected subjects with well-controlled viremia have a greater proportion of Classical (Mono1, CD14++CD16-) monocytes that produce high levels of IL-1β

When considering total monocyte percentages (all subsets combined) the HIV-infected group had a reduced frequency of total monocytes relative to total live PBMC, with a median 4.5% versus 11.3% for the HIV-uninfected group (p<0.0001). However, we did not observe this difference when comparing total monocyte counts from freshly drawn whole blood, as measured by a clinical laboratory.

**Figure 4:**
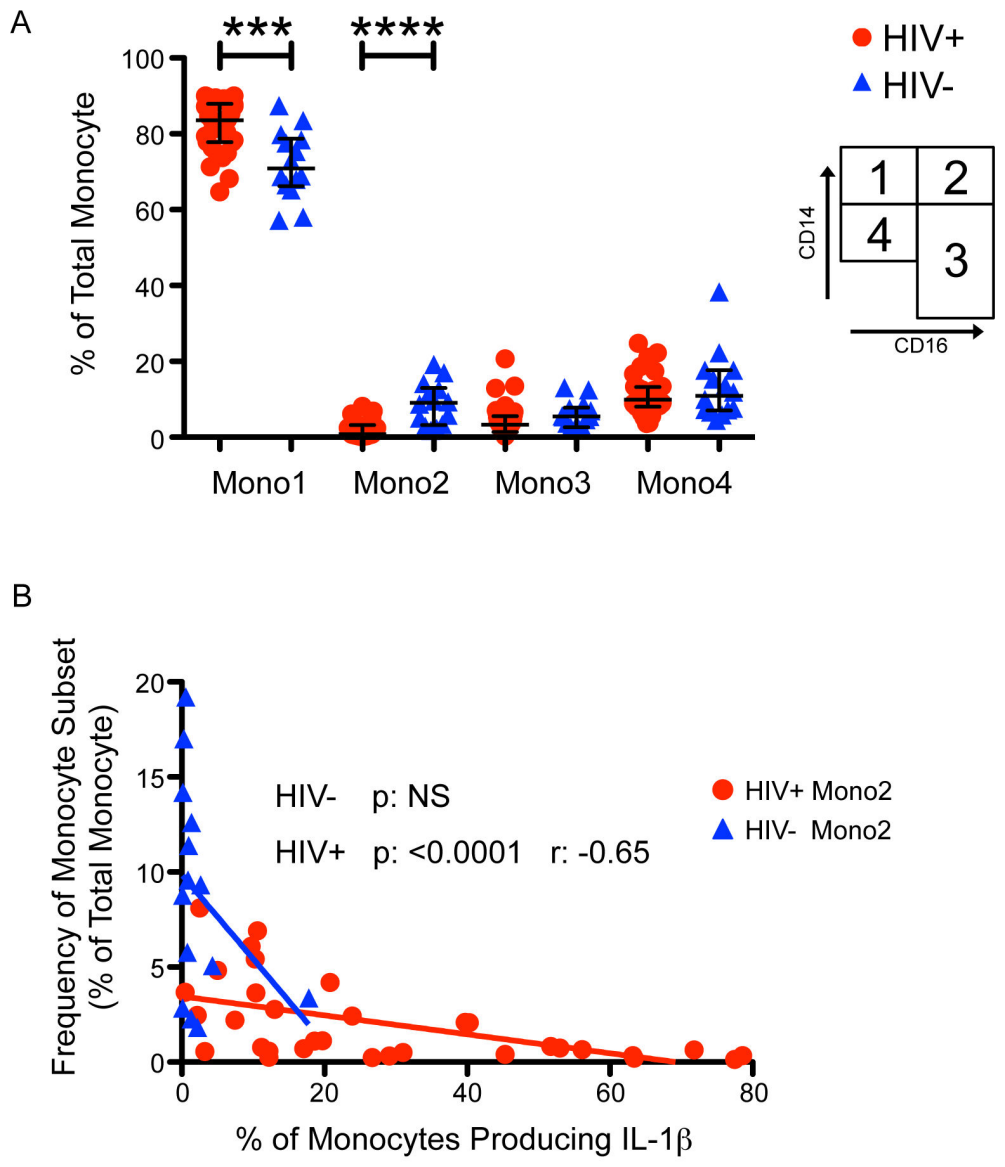
HIV-1 infected subjects with well-controlled viremia have a greater proportion of classical (Mono1, CD14++CD16-) monocytes and a lower frequency of intermediate (Mono2, CD14++CD16+) monocytes, which correlates with production of IL-1β. HIV-infected subjects are shown in red circles, and HIV-uninfected subjects are shown in blue triangles. Panel A. HIV-infected subjects had a greater fraction of monocytes that fall into the Mono1 (CD14++CD16- classical) subset, and a lower fraction that fall into the Mono2 (CD14++CD16+ intermediate) subsets. Upper right diagram represents monocyte gating scheme (Mono 1-4). Panel B. A lower proportion of monocytes in the Mono2 subset was associated with higher basal IL-1β production. **** p < 0.0001, *** p < 0.001,.

Interestingly, HIV-infected subjects had a greater proportion of classical monocytes (CD14++CD16- Mono1) (83.6% (77.9-87.9) versus 70.9% (66.2-78.8)) (Figure 4A) in the basal state. In turn, they had a much lower frequency of intermediate monocytes (CD14++CD16+ Mono2) (0.8% (0.5-3.2) versus 9% (3.2-13)) (Figure 4A). A lower proportion of monocytes in the Mono2 gate was associated with higher basal IL-1β production, p: <0.0001 (Figure 4B).

Our assay employed PBMC that had been isolated from the blood, then subsequently cryopreserved, thawed and cultured overnight. The monocyte populations seen here are relevant to *in vitro* cytokine production and are being compared with similarly treated HIV-uninfected samples. However, these populations are not necessarily reflected in fresh whole blood samples as the culture and secretion of cytokines (such as IL-10 [21]) into the media by monocytes or other cell types may have shifted the monocyte subsets.

### The basal frequency of IL-1β or IL-8 expression by monocytes predicts IL-6 production upon stimulation by LPS or oxLDL in the HIV-infected group

**Figure 5:**
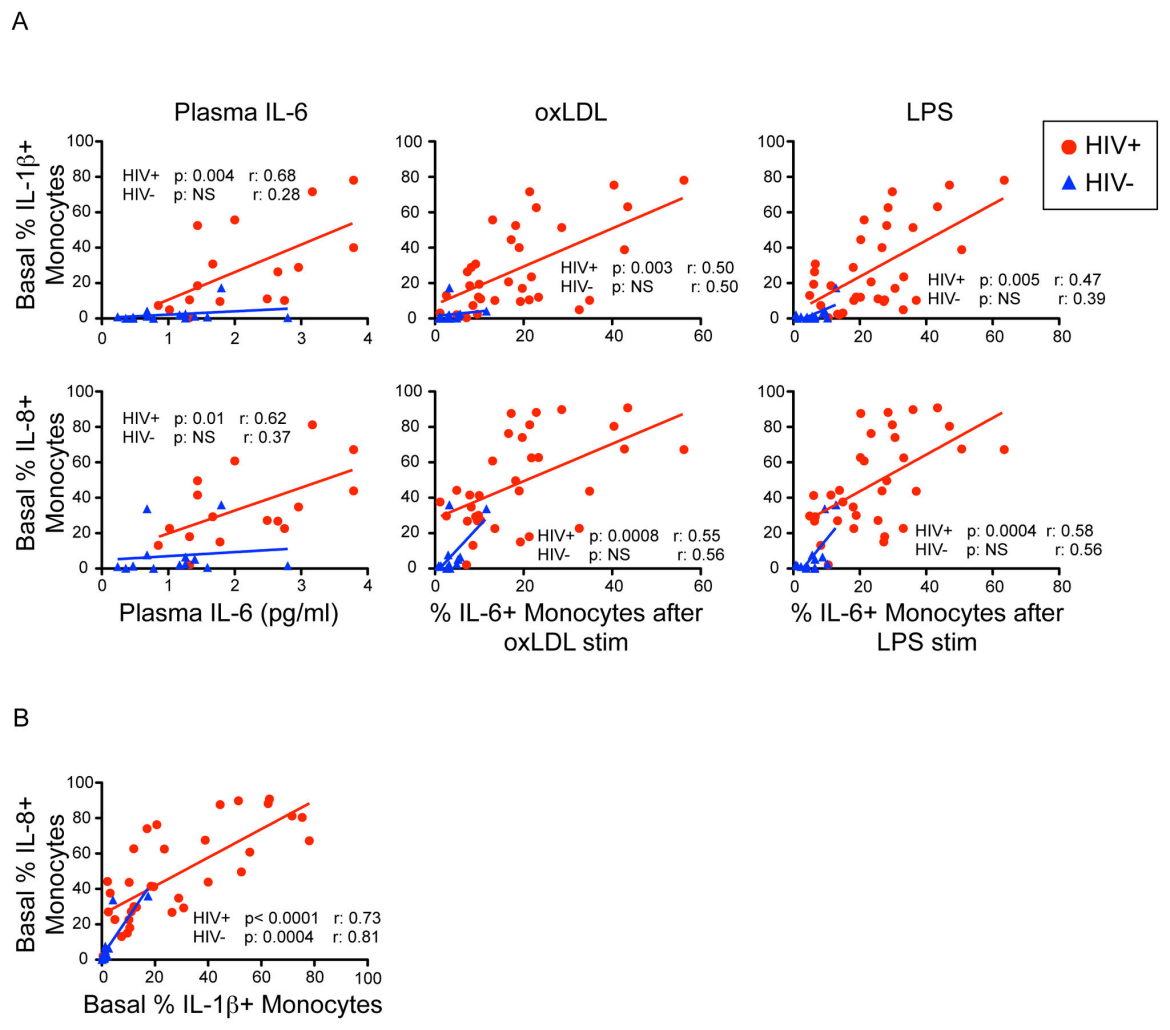
High basal IL-1β and IL-8 predict high plasma IL-6 levels and a high IL-6 response to oxLDL and LPS. HIV-infected subjects are shown in red circles, and HIV-uninfected subjects are shown in blue triangles. Panel A. In HIV-infected subjects, high basal IL-1β (upper row) or high basal IL-8 (lower row) in total monocytes was associated with higher plasma IL-6 (left column). Higher levels of IL-1β and IL-8 production in the basal state predicted high levels of IL-6 response after stimulation by either oxLDL (middle column) or LPS (right column). Exact p-values shown, NS indicates higher than the Bonferroni adjusted p-value threshold of p = 0.0133 (0.05/3 comparisons per cytokine). Panel B. Association between basal production of IL-1β and basal production of IL-8 of total monocytes.

Thus far, our experiments demonstrated that the HIV-infected group had high basal frequencies of IL-1β+ and IL-8+ monocytes, likely reflecting ongoing *in vivo* inflammation. We also observed high monocyte IL-6 production induced upon stimulation with either oxLDL or LPS. Since IL-1β has been previously shown to induce IL-6 production in peripheral blood monocytes [11], we looked to see if there was a correlation between the frequency of IL-1β+ (and IL-8+) cells in the unstimulated condition with IL-6 (Figure 5). To do so, we used either IL-6 plasma levels measured by multi-analyte bead assay or the frequency of IL-6+ monocytes upon stimulation with either oxLDL or LPS.

In Figure 5A we display the relationship of basal monocyte IL-1β or basal monocyte IL-8 to IL-6 plasma levels and monocyte responses. In HIV-infected subjects, the frequency of basal (unstimulated) IL-1β expression by total monocytes was highly correlated with plasma IL-6 levels (p = 0.004, r = 0.68) and with the frequency of IL-6 expression after stimulation by oxLDL (p = 0.003, r = 0.50) and LPS (p = 0.005, r = 0.47). The frequency of IL-8 expressing monocytes in the unstimulated condition also correlated with plasma IL-6 levels (p = 0.01, r = 0.62) and with the frequency of IL-6 expressing cells from the oxLDL condition (p = 0.0008, r = 0.55) and the LPS condition (p = 0.0004, r = 0.58) for the HIV+ group. In the HIV-uninfected group there was a trend towards higher basal IL-1β or IL-8, and higher IL-6 production, however these correlations were not significant (Figure 5). Basal expression and production of IL-1β and IL-8 were highly correlated to each other for both the HIV-uninfected (p = 0.0004, r = 0.81) and HIV-infected (p <0.0001, r = 0.73) groups (Figure 5B).

### Per cell expression of TNF is higher in ‘Non-Classical’ (CD14+ CD16+) than ‘Classical’ monocyte subsets (CD14++CD16-)

**Figure 6:**
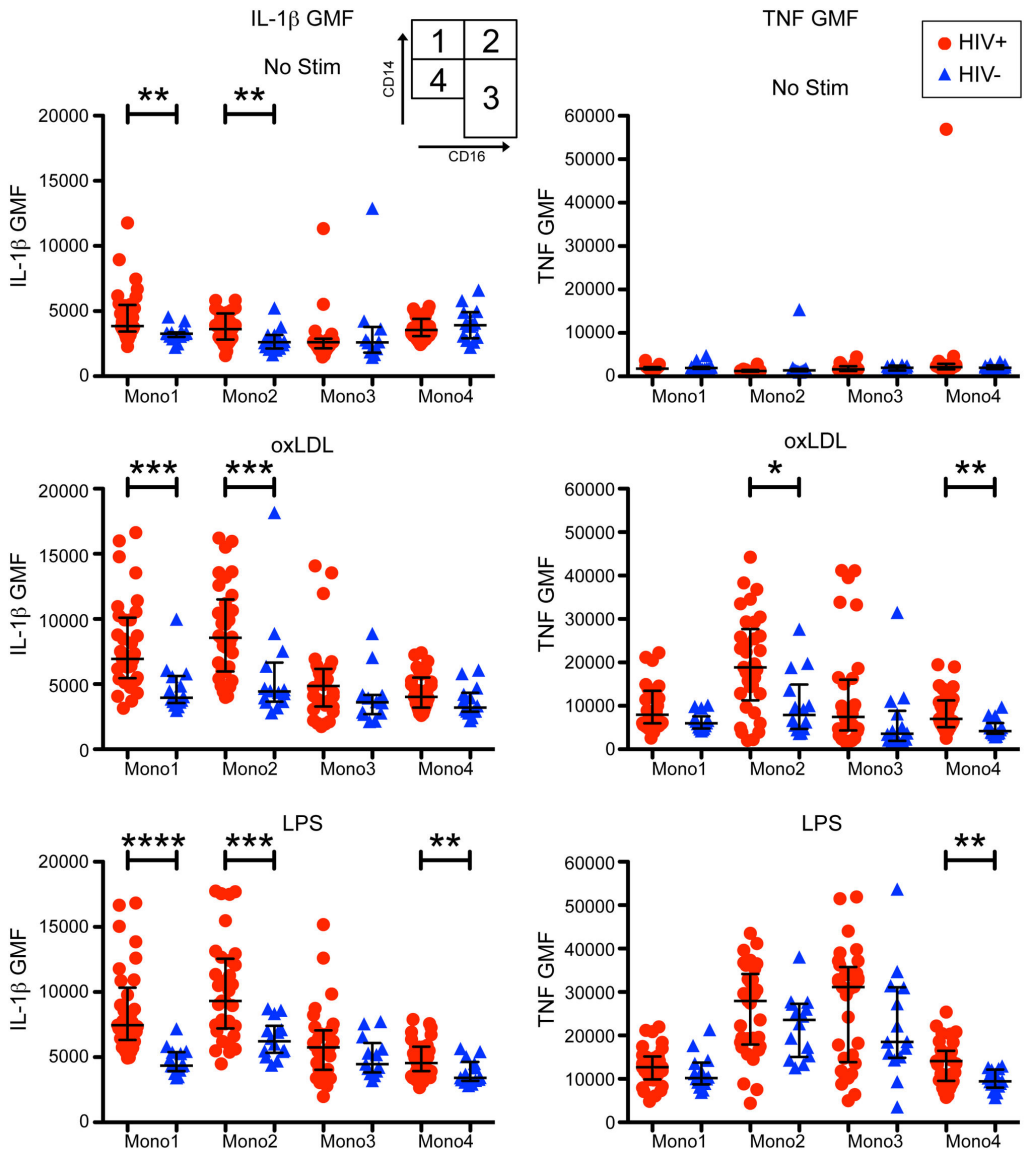
Per cell expression levels of IL-1β and TNF by each monocyte subset before and after stimulation. Geometric mean fluorescence (GMF) intensities are shown by monocyte subset for IL-1β (left column) and TNF (right column) in the basal (top row) state, and after oxLDL (middle row) or LPS (bottom row) stimulation. In HIV-infected subjects, IL-1β was expressed at highest levels by the Mono1 (CD14++CD16-) and Mono2 (CD14++CD16+) populations. TNF expression was observed in all subsets after stimuli, with the brightest monocyte subsets being Mono2 (CD14++CD16+) and Mono3 (CD14+ CD16+). Statistical significance was adjusted for multiple comparisons (p = 0.05/4 comparisons per stimulation condition: p = 0.0125). **** p < 0.0001, *** p < 0.001, ** p < 0.01, * p < 0.0125.

We evaluated the level of per cell cytokine expression using geometric mean fluorescence intensity (GMF). We found evidence that certain monocyte subsets expressed greater levels of cytokines on a per cell basis (Figure 6). Under all stimulation conditions, the Mono1 and Mono2 subsets exhibited higher per cell expression of IL-1β in HIV-infected subjects compared to HIV-uninfected. Similarly, upon stimulation with either oxLDL or LPS, several monocyte subsets from HIV-infected subjects produced more TNF on a per cell basis compared to HIV-uninfected subjects. Within the HIV-infected subjects, we observed that Mono2 and Mono3 subsets produced the highest per cell TNF expression, while Mono1 and Mono2 subsets produced the highest IL-1β. Taken together, our results suggest that HIV infection, even in the context of ART, increases per cell intensity of individual cytokine expression in specific monocyte subsets.

## Discussion

In chronically HIV-1 infected adults with stably suppressed viremia and CVD risk factors, we observed very high basal expression of the inflammatory gatekeeper cytokine IL-1β in monocytes. Upon stimulation by LPS or oxLDL, IL-1β enriched monocytes from HIV-infected adults mounted massive IL-6 responses, compared to modest responses in HIV-uninfected controls. HIV-uninfected subjects expressed little to no IL-1β in monocytes in the basal state, indicating that features of chronic HIV infection lead to a persistent stimulation of the IL-1β response in monocytes in peripheral blood.

We observed that basal production of IL-1β was highly correlated to basal production of IL-8. In addition, we observed strong increases in IL-8 production after stimuli with oxLDL or LPS. IL-8 is a particularly interesting inflammatory actor due to its very wide and finely tuned dynamic range. In healthy tissues, IL-8 is not or only weakly detectable, but may be rapidly induced by ten- to 100-fold in response to pro-inflammatory cytokines, such as IL-1, bacterial or viral products, and cellular stress. Multiple points of control over IL-8 expression allows monocytes to rapidly increase and fine-tune the amount of secreted IL-8 [22]. Therefore, IL-8 may serve as a barometer of inflammation, offering a sensitive read-out for the pro-inflammatory gatekeeping effects of IL-1β. Indeed, in our study, basal IL-8 correlated more tightly with IL-6 production upon stimulation as compared to IL-1β, possibly due to this amplification and fine-tuning.

IL-1β expression was associated with a lower fraction of monocytes of the intermediate CD14++CD16+ phenotype (Mono2). In accordance, we observed a shift towards the Mono1 phenotype in HIV+ subjects, and a lower proportion of monocytes in the Mono2 pool compared to HIV-uninfected subjects. At least one report [23] has observed that an elevation of CD14++CD16- (Classical or Mono1) monocytes predicted cardiovascular events in HIV-uninfected persons. In our study, a bias towards the classical monocyte (Mono1) phenotype in persons with well-controlled HIV infection and CVD risk was further associated with much higher levels of polyfunctional inflammatory responses, with a median 20% of all monocytes simultaneously expressing up to all 4 studied cytokines (IL-1β, IL-8, IL-6 and TNF) in HIV-infected adults compared to well below 5% in controls. The single largest source of IL-6 came from polyfunctional cells that were also producing IL-1β, IL-8 and TNF.

Our observation that TNF is produced to a greater extent on a per cell basis by Mono2 and Mono3 cells in response to stimuli is consistent with reports by Dutertre [24] Belge [25,26] and Schlitt [27] of high TNF production by CD14+ CD16+ expressing monocyte subsets. However, when considering the relative size of the monocyte subsets and their respective contribution to the total TNF produced in our assay, it was apparent that the classical Mono1 subset was the major driving force of cytokine production, due to its large size relative to the other monocyte subsets. The increased TNF production in other subsets may still prove physiologically relevant in particular situations, such as the case where differential monocyte subset recruitment across tissues is involved.

Oxidized LDL was a potent stimulus of monocytes from HIV+ subjects in our study of monocyte inflammatory potential in adults with CVD risk factors. The relationship between lipid profiles and inflammation in treated HIV-infected subjects may be complex. Indeed, results from the SMART study suggest that the treatment of HIV viremia was associated with reduction in LDL levels [5], however abnormalities remained, such as decreased cholesterol efflux and high accumulation of lipid in blood monocytes [28]. High level accumulation of lipid within monocytes may trigger internal stress pathways, pre-disposing circulating monocytes to IL-1β production. Lipid accumulation may also be responsible for intracellular or genomic changes leading to ‘poised’ epigenetic states [29] and massive responses to common triggers like oxLDL and LPS. Indeed, we found that higher HDL levels in the blood of HIV+ subjects, measured several ways, were associated with higher basal IL-1β levels in monocytes (data not shown). Inflammation-induced dysfunctional HDL has been previously described [30] and our results also suggest that HDL levels in the context of HIV infection may serve as a pro-inflammatory agent, perhaps via IL-8 mediated oxidation of HDL in the blood by neutrophil recruitment and myeloperoxidase release.

A recent trial of Rosuvastatin in adults with well-controlled HIV [31] suggested high IL-6 but not expansion of monocyte subsets (Mono 1, 2 or 3), was associated with higher carotid intima media thickness (cIMT). Our results likewise found evidence for high IL-6 in patients with well-controlled HIV viremia with CVD risk factors. At least one study has concluded that HIV is not a driver of IL-6 [32], which was based on an observed lack of association of HIV RNA levels with IL-6. The SUN study [33] of HIV infection, inflammation and progression to cardiovascular clinical marker endpoints, reported an association of monocyte phenotype profile (higher proportions of CD14++CD16+ intermediate monocytes (Mono2)). This intermediate monocyte phenotype (Mono2) associated with progression in coronary arterial calcification (CAC) score over a 2 year period of follow-up. Notably, a lower fraction of the SUN study participants, only 78%, were reported to be on HAART during the study, with only 72% exhibiting a viral load less than 400 copies/ml. By contrast, all of our study population was on HAART, 96% had a viral load less than 48 copies/ml, and all were less than 400 copies/ml at time of study. Hence, our results, while not part of a study of predictors of CVD clinical surrogates, such cIMT or CAC, may differ with respect to observed phenotypes. This effect may be due to differences in the populations studied. That said, the focus of our study was not phenotypes, but to explore the inflammatory responses of monocytes ex vivo from treated, chronically HIV-infected adults at risk for CVD.

In chronically HIV-1 infected persons with well-controlled viremia and CVD risk factors, circulating blood monocytes enriched for mature IL-1β expression in the absence of stimulation are a major source of IL-6 and other inflammatory cytokines upon stimulation. HIV-1 negative, age matched adults with similar or modestly improved CVD risk factors did not show expansion of an IL-1β enriched monocyte population, nor the high IL-6 responses to LPS and oxLDL stimuli. While we found a shift towards a Mono1 (Classical) phenotype in HIV-infected adults, we also found that Mono2 and Mono3 populations expressed the highest levels of TNF, and importantly observed significantly higher per cell IL-1β and TNF expression intensities in HIV-infected adults, suggesting a continuing role for TNF in morbidity during treated HIV infection. The elevated risk for CVD in HIV-infected persons may be conferred at least in part by hyper-inflammatory, IL-1β enriched monocytes that mount high level IL-6, IL-8 and TNF responses to triggers such as LPS and oxLDL that are commonly found in the blood of older, chronically HIV-1 infected adults. IL-1β enriched monocytes may be a major source of blood plasma IL-6 in treated HIV infection, and hence be an effective point of intervention. Further studies will be required to determine if IL-1β loaded, highly inflammatory monocytes will be effective cellular biomarkers of CVD in HIV disease, or represent an element in the pathology of HIV all-cause mortality, CVD pathogenesis, or other HIV-associated morbidities (such as renal impairment or neurocognitive decline) during long-term anti-retroviral treatment.
